# Impacts of Drought on Maize and Soybean Production in Northeast China During the Past Five Decades

**DOI:** 10.3390/ijerph17072459

**Published:** 2020-04-03

**Authors:** Chunyi Wang, Hans W. Linderholm, Yanling Song, Fang Wang, Yanju Liu, Jinfeng Tian, Jinxia Xu, Yingbo Song, Guoyu Ren

**Affiliations:** 1 State Key Laboratory of Severe Weather, Chinese Academy of Meteorological Sciences, Beijing 100081, China; songyl@cma.gov.cn (Y.S.); fangw@cma.gov.cn (F.W.); 2 Department of Earth Sciences, University of Gothenburg, 405 30 Gothenburg, Sweden; hansl@gvc.gu.se; 3 Department of Geography, University of Cambridge, Cambridge CB2 3EN, UK; 4 National Climate Center, China Meteorological Administration, Beijing 100081, China; liuyanj@cma.gov.cn (Y.L.); guoyoo@cma.gov.cn (G.R.); 5 Faculty of Agricultural and Nutritional Sciences, Kiel University, 24118 Kiel, Germany; stu115178@mail.uni-kiel.de; 6 Climate Center of Sichuan Province, China Meteorological Administration, Chengdu 610072, China; caiwy@cma.gov.cn; 7 National Meteorological Center, China Meteorological Administration, Beijing 100081, China; songyb@cma.gov.cn; 8 Department of Atmospheric Sciences, School of Environmental Studies, China University of Geosciences, Wuhan 430074, China

**Keywords:** drought, climate change, maize and soybean, northeast China

## Abstract

Climate change has a distinct impact on agriculture in China, particularly in the northeast, a key agriculture area sensitive to extreme hydroclimate events. Using monthly climate and agriculture data, the influence of drought on maize and soybean yields—two of the main crops in the region—in northeast China since 1961 to 2017 were investigated. The results showed that the temperature in the growing season increased by 1.0 °C from the period 1998–2017 to the period 1961–1980, while the annual precipitation decreased slightly. However, precipitation trends varied throughout the growing season (May–September), increasing slightly in May and June, but decreasing in July, August and September, associated with the weakening of the East Asian summer monsoon. Consequently, the annual and growing season drought frequency increased by 15%, and 25%, respectively, in the period 1998–2017 relative to the period 1961–1980. The highest drought frequency (55%) was observed in September. At the same time, the drought intensity during the growing season increased by 7.8%. The increasing frequency and intensity of drought had negative influences on the two crops. During moderate drought years in the period 1961–2017, 3.2% and 10.4% of the provincial maize and soybean yields were lost, respectively. However, during more severe drought years, losses doubled for soybean (21.8%), but increased more than four-fold for maize (14.0%). Moreover, in comparison to the period 1961–1980, a higher proportion of the yields were lost in the period 1998–2017, particularly for maize, which increased by 15% (increase for soybean was 2.4%). This change largely depends on increasing droughts in August and September, when both crops are in their filling stages. The impact of drought on maize and soybean production was different during different growth stages, where a strong relationship was noted between drought and yield loss of soybean in its filling stage. Given the sensitivity of maize and soybean yields in northeast China to drought, and the observed production trends, climate change will likely have significant negative impacts on productivity in the future.

## 1. Introduction

Droughts are major natural disasters that can affect large areas. Severe droughts can have substantial effects on economic, agricultural, ecological, and environmental activities [1,2,3]. Across the world, drought is one of the most serious environmental stresses affecting plant growth and development and, consequently, the yields and productivity of crops [4]. For instance, it was estimated that during the period 2000–2007, approximately 3% of the global cereal production was lost to extreme drought disasters [5]. In China, drought is one of the main natural hazards, occasionally causing crop failures. Agricultural drought disasters are important stress factors that affect the sustainable development of agriculture and environment in China [6]. In particular, northeast China, where the onset of the summer monsoon occurs late in the summer, is frequently affected by droughts. As an example, during June to August 2014, precipitation was 20–50% below normal in northeast China, resulting in decreased maize (*Zea mays* L.) production by 3.93 million tons in the Liaoning province [7].

Northeast China, including the Heilongjiang, Jinlin and Liaoning provinces, is a main agriculture area in China. Maize, rice (*Oryza sativa* L.), and soybean (*Glycine max* (L.) Merrill) are the most important crops, with a total farming area of nearly 25.0 million hectares in 2018 (15.0% of total China’s farming area) [8]. Furthermore, maize and soybean production in 2018 was 84.4 and 7.6 million tons, respectively, corresponding to approximately 32% of the total maize production and approximately 40% of the total soybean production in China [8]. Given the importance of these two crops, and the negative influences of drought on their yields [9], a comprehensive understanding of the impact of drought is vital when evaluating how climate change will affect their production.

To predict, monitor, and assess the severity of droughts, several numerical drought indices have been developed [10,11,12,13,14,15,16,17,18,19,20]. In 2009, the WMO (World Meteorological Organization) recommended that the standardized precipitation index (SPI) [12], which is calculated only from precipitation, should be the main meteorological drought index used to monitor and follow up the evolution of drought conditions [21]. The Palmer drought severity index (PDSI) was originally developed for North American conditions by the United States Weather Bureau in 1965 [10] and is one of the most widely used drought indices worldwide. The standardized precipitation and evapotranspiration index (SPEI) takes into account both precipitation and potential evapotranspiration. So, unlike the SPI on which it is based, the SPEI captures the impact of increased temperature on water demand [3]. The impacts of changes in drought patterns on different spatial scales have been well studied [22,23,24,25,26,27,28,29,30,31]. However, less attention has been paid to how droughts affect crops during different growth stages.

To better understand how droughts affect crop production, it is vital to explore the different growth stages [32]. For example, droughts have more impact during the filling stage than during the seedling stage [33]. In this paper, we present results on the impact of drought on maize and soybean during different growth stages in northeast China in recent decades.

## 2. Material and Methods

### 2.1. Study Area

**Figure 1:**
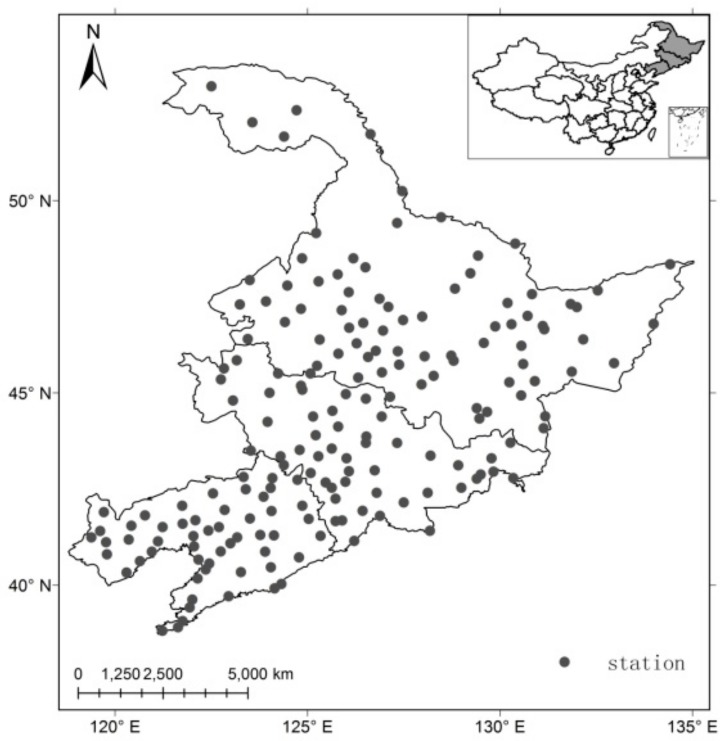
The distribution of weather stations in the northeast China.

In this study, northeast China is defined as the area 48° N–55° N and 118° E–135° E, and includes the provinces of Heilongjiang, Jilin and Liaoning, but excludes the eastern part of Inner Mongolia (Figure 1). The climate of northeast China can be characterized mainly as a temperate monsoon climate. The annual mean surface air temperature is only 5.4 °C (1981 to 2010), which is 7.8 °C lower than that of China. The total annual precipitation generally ranges from 370 to 1000 mm. During the growing season (from May to September), the mean surface air temperature is 18.8 °C, and the average total precipitation is 487 mm.

### 2.2. Data

In order to investigate growing season droughts in northeast China in recent decades, monthly precipitation, mean temperatures, and minimum and maximum temperatures for the period 1961–2017 were used, provided by the China Meteorological Administration. Only station series covering periods longer than 57 years with ≤5% of missing data were chosen (Figure 1). In total, 183 stations were included in the study.

Particular attention was paid to the negative impacts of drought on maize and soybean production, and so the provincial-level yields of maize and soybean were applied by the Ministry of Agriculture of the People’s Republic of China from 1958 to 2017. The yield loss was calculated over the period 1961–2017, when there was an overlap between climate and yield loss data.

### 2.3. Methods

#### 2.3.1. The Drought Index

Since the standardized precipitation and evapotranspiration index (SPEI) takes into account both precipitation and potential evapotranspiration, the monthly and annual SPEI indices calculated from the station data were used to analyze regional drought events. The SPEI uses the standardized precipitation index as a basis but includes a temperature component that allows the index to account for the impact of temperature on drought development through a basic water balance calculation. The SPEI has an intensity scale in which both positive and negative values are calculated, identifying wet and dry events. The SPEI was calculated following the classical approximation [3]:

(1) $$SPEI=W-\frac{C_{0}+C_{1}W+C_{2}W^{2}}{1+d_{1}W+d_{2}W^{2}+d_{3}W^{3}}$$

where $W=\sqrt{-2\ln\left(1-P\right)}$ for *p* ≤ 0.5 and *P* is the probability of exceeding a determination d value, *P* = 1 − *F*(*x*), and *Fx* is the probability distribution of the cumulative water deficit series. If *p* *>* 0.5, then *p* is replaced by 1 − *p* and the sign of the resultant SPEI is reversed. The constants are c_0_ = 2.515517, c_1_ = 0.802853, c_2_ = 0.010328, *d*_1_ = 1.432788, *d*_2_ = 0.189269, and *d*_3_ = 0.001308. In this study, potential evapotranspiration in the SPEI is deduced with the Penman–Monteith equation [34].

#### 2.3.2. The Yield Loss Rate

In northeast China, maize can be cultivated from May to September. It is usually sowed in May, with a seeding stage in June, fast growth in July, flowering in August, and grain filling in September. Soybean is also cultivated from May to September; it is sowed in May, but the grain filling stage is generally in August, and it matures in September. Yields of maize and soybean are mainly influenced by technological development (e.g., variety change and fertilization application) and climate, while yield losses are induced mainly by extreme weather events (droughts, low temperature, heat waves), as well as plant diseases and insect pests. In China, the variety of crops changed in general every 3–5 years [35]. The yield loss rate in a specific year was calculated relative to the maximal yield during the last 5 years. The yield loss rate was defined as follows:

(2) $$Y_{l}=\frac{Yi-Ym}{Ym}\times 100\%$$

Y_l_: The yield loss rate, induced mainly by climate events,

Yi: Yield in a given year, and

Ym: Maximal yield during the last 5 years.

## 3. Results

### 3.1. Changes in Drought Characteristics

As temperature and precipitation are the most important factors for the growth of crops, and precipitation deficit, as well as high temperatures, can induce droughts [9], we first investigated changes in those parameters.

**Figure 2:**
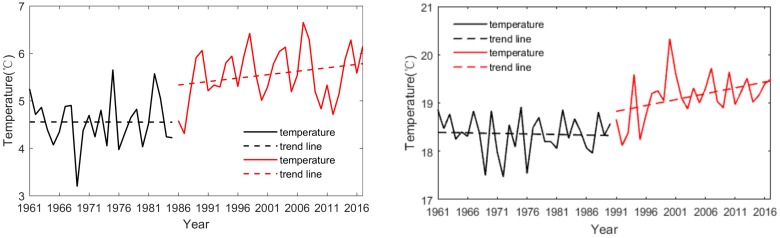
The change in annual mean surface air temperature (**left**) and growing season temperature (**right**) in northeast China during the period 1961–2017.

The temperature in northeast China is lower than that in other farming areas in China due to its high-latitude environment. Here, maize and soybean usually grow from May to September. Average (1981–2010) temperatures were 14.8 °C in May, 20.0 °C in June, 22.6 °C in July, 21.5 °C in August, and 15.2 °C in September. The annual mean surface air temperature (SAT) averaged for the studied region displays large inter-annual variability, and the mean temperatures are different between 1961–1985 and 1991–2017. The temperature was quite stable in the period 1961–1985 but increased from 1986 to 2017 (Figure 2). The annual mean temperature was 5.6 °C during the last two decades, which is 1.1 °C higher than that during the period 1961–1980. The same was observed for growing season temperatures, which increased by 1.0 °C in the period 1998–2017 compared to the period 1961–1980. The influence of temperature on crop growth is complex: at these relatively high latitudes, a warm growing season favors crop growth, but high temperatures (for example, daily maximum temperature > 30 °C) in combination with precipitation deficits causing drier soils in summer can have negative effects on maize during flowering stage [36].

**Figure 3:**
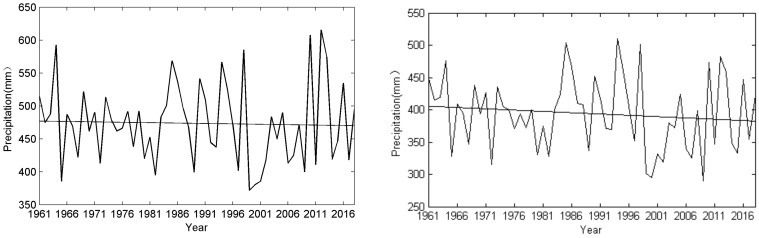
Change in annual total precipitation (**left**) and growing season precipitation (**right**) in northeast China during the period 1961–2017.

Total precipitation was 50.7 mm in May, 90.3 mm in June, 155.6 mm in July, 134.4 mm in August, and 55.9 mm in September (1980–2010). Clearly, precipitation during parts of the maize and soybean growing season is quite low. Precipitation during the growing season decreased slightly by 5% in the period 1998–2017 compared to the period 1961–1980 (Figure 3). Precipitation displayed different changes in different months; precipitation increased slightly in May and June from the period 1998–2017 to the period 1961–1980, which is the pre-monsoon season, but decreased in July, August and September (Table 1). In northeast China, droughts induced by a combination of low precipitation and high temperatures frequently occur in spring and autumn, and so increasing precipitation in early summer has positive impacts on maize and soybean growth, while the decreasing precipitation in autumn causes increased drought pressure on the crops.

At the end of spring and during the beginning of summer (May to June), precipitation in the studied region is mainly affected by the cold vortex over northeast China [37]. From midsummer to the early autumn (July to September), when the front of the East Asian summer monsoon moves northward to northeast China, the amount of precipitation is closely related to the strength of the monsoon and its water vapor transport [37,38]. Over the past 60 years, increased precipitation in late spring/early summer in northeast China is consistent with an increasing trend of northeast cold vortex frequency [37]. The decrease in summer to early autumn precipitation is, on the other hand, closely related to the weakening of the East Asian summer monsoon associated with global warming [38], where less water vapor is transported to the northeast regions of China [39].

**Figure 4:**
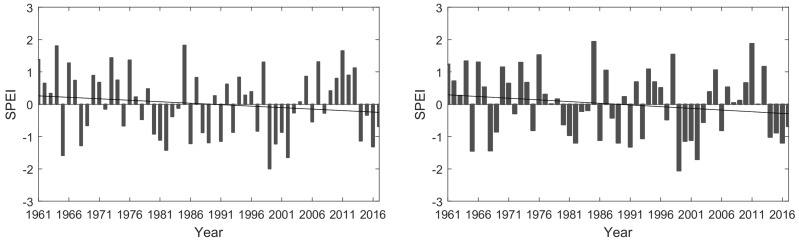
Change in the annual standardized precipitation and evapotranspiration index (SPEI) (**left**) and growing season (May–September) drought index (**right**) during the period 1961–2017.

Droughts increased during the studied period in northeast China. In this study, droughts were defined as an SPEI below −0.5, being severe when the SPEI was less than −1. Maize and soybean growing season drought frequency (defined as number of drought years/decade) increased during the study period, from 30% in the 1960s, 20% in the 1970s, 40% in the 1980s and 1990s, to 47% in 2001 to 2017, showing strong decadal variability. Growing season drought frequency increased by 25% in the period 1998–2017 relative to the period 1961–1980 (Figure 4). Looking at monthly data, drought frequency decreased in early summer (May and June) due to increasing precipitation, while the opposite was seen in July to September (Table 1). Drought frequency in September amounted to 55% in the period 1998–2017 which was an increase by 25% from the period 1961–1980. Thus, there was a risk of September drought in more than half of years, likely induced by the weakening East Asian summer monsoon under global warming. Moreover, the drought intensity increased during the study period. The annual mean (growing season) SPEI was −1.2 (−1.1) during the period 1998–2017, which was an increase by 26.9% (7.8%) relative to the period 1961–1980. That both drought frequency and intensity have increased in northeast China is in line with previous research [31,40]. Further, our results indicate an increased frequency of drought mainly outside the growing season, which may be linked to an earlier onset of the growing season in northern China [41]. An issue to take into consideration in the analysis is the urbanization effect on estimated temperature trends in recent decades in this region. Previous works have suggested that there is a significant urbanization effect on average annual and seasonal mean temperatures, resulting in an overestimate of the regional temperature trends [42,43]. This may have resulted in positive biases in calculated potential evapotranspiration and needs to be further investigated in future research.

### 3.2. The Influence of Drought on Crops

The yields of maize and soybean showed a steady long-term increase from 1961 to 2017. The mean maize yield in the 1960s of 1476.3 kg/ha increased to 6314.1 kg/ha in the 2000s in Jilin province, an increase of 4.3-fold from the 1960s to the 2000s. In the Liaoning and Heilongjiang provinces maize yields increased by 2.8-fold over the same time. The mean soybean yield in the 1960s of 945.6 kg/ha increased to 2156.8 kg/ha in the 2000s in Liaoning province, an increase of 2.3-fold from the 1960s to the 2000s. In the Heilongjiang and Jilin provinces, the increases were 1.4- and 2.5-fold, respectively. These increasing trends of yield were mainly due to technological development (e.g., maize and soybean variety changes and fertilization application). Any production loss was mainly caused by various disasters, such as drought and low temperatures as well as plant diseases and insect pests, during the growing period.

Drought can seriously influence maize and soybean grain yields negatively, e.g., by affecting stomatal conductance, photosynthesis, leaf expansion, and progression in the plant cycle [44]. However, short periods of drought may have some positive effects. For instance, soybean plants can show physico-biochemical growth compensations for loss and damage caused by drought stress when rehydrated [45], and water stress can initially lead to an increase in shoot length, which could promote uptake of water and nutrients. However, if droughts continues for extended periods of time, the water content, root activity, chlorophyll content of leaves, soluble protein and biomass of the crops are reduced.

**Figure 5:**
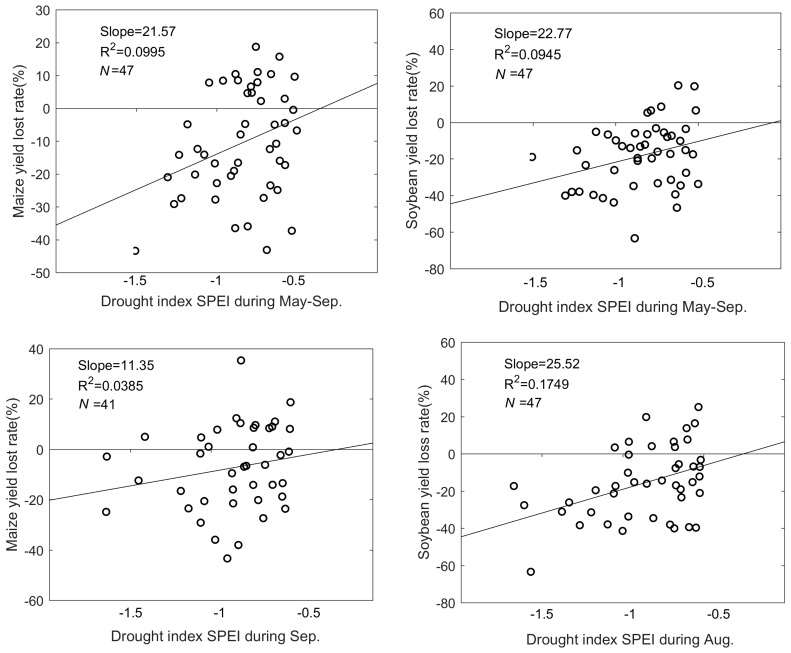
The association between the yield loss rate of maize and soybean and the drought index during May–September, September and August.

The provincial yields of maize and soybean in northeast China are sensitive to drought. In 68% of the years with dry growing seasons (SPEI < −0.5) in the period 1961–2017, maize yields were reduced, and the reduction was related to the severity of the drought: there was an average loss of 3.2% of provincial maize yields in years with moderate drought (SPEI = −0.5) during the growing season, but this increased to 14.0% during severe drought (SPEI = −1.0) years (Figure 5). The effect of drought on maize yields increased through time: the average maize production reduction in dry years was 15.6% during the period 1998–2017, which was an increase by 15% from the period 1961–1980. The influence of drought on soybean, on the other hand, was more serious. Soybean production decreased in 87.2% of the years, with a growing season SPEI < −0.5. Further, for soybean, the yield loss increased with a lower growing season SPEI: 10.4% and 21.8% of the provincial yields were lost during moderate and severe drought years, respectively, in the period 1961–2017 (Figure 5). In the period 1998–2017, the average soybean production loss during dry years was 20.4%, which was an increase by 2.4% from the period 1961–1980. Yield losses are influenced by many factors, such as climate factors, plant diseases and insect pests, and so it is important to apply a method that removes the non-climatic effects.

In northeast China, maize is cultivated from May to September, and grain filling occurs in September. The grain filling stage of soybean is generally in August, and it matures in September. Because the water storage capacity of the two crops differs among growth stages, the timing of drought is of importance. For instance, if there is sufficient precipitation after a drought in the seedling stage, later growth can make up for the lack of biomass in the early stage of the crops [9]. Therefore, drought in the seedling stage of crops usually has limited effects on yields. Both maize and soybean are, on the other hand, very sensitive to water deficit during the filling period [33], which mainly occurs in August and September for northeast China. During drought, the water potential of the leaves begins to decline, and then the photosynthetic rate decreases. Additionally, the response of the development of the reproductive organs to drought stress is more sensitive than that of the vegetative organs. Drought during the grain filling stage reduces the grain storage capacity, resulting in decreased grain weight and yield [33], and the negative impacts of drought during the grain-filling period are irreparable. So, drought in September can have great impacts on maize production, while drought in August likely has larger negative impacts on soybean production.

Our results showed that maize production decreased in 63.4% of years when drought (SPEI < −0.5) occurred in September. For soybean, yields decreased in 77.3% of the years with droughts (SPEI < −0.5) in August. The correlation between maize production and the SPEI was 0.32 (*p* < 0.05) in May–September. Significant correlations between soybean production and the SPEI were found in May–September (*r* = 0.31, *p* < 0.05) and August (*r* = 0.42, *p* < 0.005). In the other months, correlations were small and non-significant. The results indicate a stronger relationship between drought and yield loss for soybean in its filling stage compared to maize.

## 4. Conclusions

Maize and soybean are the main crops in northeast China, whose productions are sensitive to weather conditions during the growing season (May to September), particularly the latter part. Using monthly climate and agriculture data, the influence of weather, specifically drought, on maize and soybean production in northeast China in 1961 to 2017 was investigated. Associated with the weakening of the East Asian summer monsoon, an increasing annual mean temperature, together with a slight decrease in annual precipitation, resulted in an increase in drought frequency and intensity, which was most pronounced in July-September. Growing season drought frequency increased by 25% (55% in September) in the period 1998–2017 relative to the period 1961–1980, but drought intensity only increased by 7.8%. The results showed that the drivers of crop failure of maize and soybean in northern China changed during the studied period. In the period 1960–1980, crop failure of maize and soybean was mainly related to low temperatures, while drought was the main reason in subsequent decades. Moreover, a higher proportion of the yields was lost in the period 1998–2017 compared to the period 1961–1980, and maize seems to have been more susceptible to increasing droughts compared to soybean.

Due to limited irrigation possibilities in most farming regions of northeast China, the production of maize and soybean is very sensitive to temperature and precipitation variations, and increasing drought frequency and intensity will have severe impacts on yields in a warmer future climate. Given the agricultural importance of northern China, it is necessary to further investigate the impact of droughts on crop yields, including more high-resolution analyses using daily data.

## Figures and Tables

**Table 1:** Change in temperature, precipitation and drought frequency (SPEI) in northeast China in the period 1998–2017 relative to the period 1961–1980.

Month	Temperature (°C)	Precipitation (mm)	Drought Frequency (%)
May	+0.9	+10.3	−5%
June	+1.0	+6.2	−15%
July	+0.5	−16.8	+5%
August	+0.7	−1.2	+10%
September	+1.2	−13.0	+25%

## References

[1] Begueria S., Vicente-Serrano S.M., Angulo-Martinez M. (2010). A multiscalar global drought dataset: The SPEIBASE A new gridded product for the analysis of drought variability and impacts. Bull. Am. Meteorol. Soc..

[2] Li B., Su H., Chen F., Wu J., Qi J. (2013). The changing characteristics of drought in China from 1982 to 2005. Nat. Hazards.

[3] Vicente-Serrano S.M., Begueria S., Lopez-Moreno J.I., Angulo M., El Kenawy A. (2010). A new global 0.5 degrees gridded dataset (1901–2006) of a multiscalar drought index: Comparison with current drought index datasets based on the palmer drought severity index. J. Hydrometeorol..

[4] Miao Z., Han Z., Zhang T., Chen S., Ma C. (2017). A systems approach to a spatio-temporal understanding of the drought stress response in maize. Sci. Rep..

[5] Lesk C., Rowhani P., Ramankutty N. (2016). Influence of extreme weather disasters on global crop production. Nature.

[6] Wang Q., Liu Y., Zhang Y., Tong L., Li X., Li J., Sun Z. (2019). Assessment of spatial agglomeration of agricultural drought disaster in China from 1978 to 2016. Sci. Rep..

[7] China Meteorological Disaster Yearbook (CMDY) (2015). China Meteorological Disaster Yearbook.

[8] China Statistical Yearbook (CSY) 9 (2019). China Statistical Yearbook, National Bureau of Statistics of China.

[9] Song Y.L., Wang J.L. (2017). The Assessment of Influence of Agro-Meteorological Disasters on Agriculture under Climate Change in China.

[10] Palmer W.C. (1965). Meteorological Drought.

[11] Tarpley J.D., Schneider S.R., Money R.L. (1984). Global vegetation indices from the NOAA-7 meteorological satellite. J. Appl. Meteorol..

[12] McKee T.B., Doesken N.J., Kleist J. (1993). The relationship of drought frequency and duration to time scales. Proceedings of the 8th Conference on Applied Climatology.

[13] Wu H., Hayes M.J., Weiss A., Hu Q. (2001). An evaluation of the Standardized Precipitation Index, the China-Z Index and the statistical Z-Score. Int. J. Climatol..

[14] Lyon B. (2014). The strength of El Niño and the spatial extent of tropical drought. Geophys. Res. Lett..

[15] Narasimhan B., Srinivasan R. (2005). Development and evaluation of Soil Moisture Deficit Index (SMDI) and Evapotranspiration Deficit Index (ETDI) for agricultural drought monitoring. Agric. For. Meteorol..

[16] Nalbantis I., Tsakiris G. (2008). Assessment of hydrological drought revisited. Water Resour. Manag..

[17] Anderson M.C., Hain C., Wardlow B., Pimstein A., Mecikalski J.R., Kustas W.P. (2011). Evaluation of drought indices based on thermal remote sensing of evapotranspiration over the continental United States. J. Clim..

[18] Woli P., Jones J.W., Ingram K.T., Fraisse C.W. (2012). Agricultural reference index for drought (ARID). Agron. J..

[19] Hao Z., AghaKouchak A. (2013). Multivariate standardized drought index: A multi-Index parametric approach for drought analysis. Adv. Water Resour..

[20] Beguería S., Vicente-Serrano S.M., Reig F., Latorre B. (2014). Standardized precipitation evapotranspiration index (SPEI) revisited: Parameter fitting, evapotranspiration models, tools, datasets and drought monitoring. Int. J. Climatol..

[21] Hayes M.J., Svoboda M., Wall N., Widhalm M. (2011). The Lincoln declaration on drought indices: Universal meteorological drought index recommended. Bull. Am. Meteorol. Soc..

[22] Sheffield J., Andreadis K.M., Wood E.F., Lettenmaier D.P. (2009). Global and continental drought in the second half of the twentieth century: Severity-area-duration analysis and temporal variability of large-scale events. J. Clim..

[23] Kim D.W., Byun H.R., Choi K.S., Oh S.B. (2011). A spatiotemporal analysis of historical droughts in Korea. J. Appl. Meteorol. Clim..

[24] Qi H.X., Zhi X.F., Bai Y.Q. (2011). Interdecadal variation and trend analysis of the drought occurrence frequency in China. Trans. Atmos. Sci..

[25] Sternberg T. (2011). Regional drought has a global impact. Nature.

[26] Wang A.H., Lettenmaier D.P., Sheffield J. (2011). Soil moisture drought in China, 1950–2006. J. Clim..

[27] Huang S.Z., Chang J.X., Huang Q., Chen Y.T. (2014). Spatio-temporal changes and frequency analysis of drought in the Wei River Basin, China. Water Resour. Manag..

[28] Vergni L., Todisco F., Mannocchi F. (2015). Analysis of agricultural drought characteristics through a two-dimensional copula. Water Resour. Manag..

[29] Wang W., Zhu Y., Xu R.G., Liu J.T. (2015). Drought severity change in China during 1961-2012 indicated by SPI and SPEI. Nat. Hazards.

[30] Zhai J., Huang J., Su B., Cao L., Wang Y., Jiang T., Fischer T. (2016). Intensity-area duration analysis of droughts in China 1960–2013. Clim. Dyn..

[31] Hou W., Feng G., Yan P., Li S. (2018). Multifractal analysis of the drought area in seven large regions of China from 1961 to 2012. Meteorol. Atmos. Phys..

[32] Prasad P.V.V., Staggenborg S.A., Ristic Z., Ahuja L.H., Ma L., Saseendran S. (2008). Impacts of drought and/or heat stress on physiological, developmental, growth and yield processes of crop plants. Responses of Crops to Limited Water: Understanding and Modeling Water Stress Effects on Plant Growth Processes.

[33] Huang Y., Li J., Wang Y., Zhang X.Y., Li X.Y. (2019). Impact simulation of drought on maize growth and yield in different growth stages. J. Agric. Catastrophol..

[34] Allen R.G., Pereira L.S., Raes D., Smith M. (1998). Crop Evapotranspiration–Guidelines for Computing Crop Water Requirements–FAO Irrigation and Drainage.

[35] Song Y.L., Wang C.Y., Ren G., Zhao Y., Linderholm H.W. (2015). The relative contribution of climate and cultivar renewal to shaping rice yields in China since 1981. Theor. Appl. Climatol..

[36] Yang F.Y., Zheng Q.H., Luo J.M., Li W.K. (2015). Practical Agro-Meteorological Index.

[37] Shen B.Z., Lin Z.D., Lu R.Y., Lian Y. (2011). Circulation anomalies associated with interannual variation of early- and late-summer precipitation in Northeast China. Sci. China Earth Sci..

[38] Sun L., An G., Tang X.L. (2003). Relationship between the Northeast Asian summer south wind anomaly and the precipitation in Northeast China. Chin. J. Atmos. Sci..

[39] Zhang J., Qian W.H., Ding T. (2010). Characteristics and trends of rainfall events in Northeast China from May to September during 1956–2008. Meteorol. Mon..

[40] Li X.Z., Zhou W., Chen Y.D. (2015). Assessment of regional drought trend and risk over China: A drought climate division perspective. J. Clim..

[41] Song Y., Linderholm H.W., Chen D., Walther A. (2010). Trends of the thermal growing season in China, 1951–2007. Int. J. Climatol..

[42] Ren G.Y., Zhou Y.Q. (2014). Urbanization effect on trends of extreme temperature indices of national stations over mainland China, 1961–2008. J. Clim..

[43] Tysa S.K., Ren G., Qin Y., Zhang P., Ren Y., Jia W., Wen K. (2019). Urbanization effect in regional temperature series based on a remote sensing classification scheme of stations. J. Geophys. Res. Atmos..

[44] Parent B., Tardieu F. (2014). Can current crop models be used in the phenotyping era for predicting the genetic variability of yield of plants subjected to drought or high temperature?. J. Exp. Bot..

[45] Dong S., Jiang Y., Dong Y., Wang L., Wang W., Ma Z., Yan C., Ma C., Liu L. (2019). A study on soybean responses to drought stress and rehydration. Saudi J. Biol. Sci..

